# Efficacy and Safety of Sitafloxacin in Treating Low-risk Febrile Neutropenia in Patients with Lung Cancer

**DOI:** 10.31662/jmaj.2021-0227

**Published:** 2022-05-23

**Authors:** Rintaro On, Takemasa Matsumoto, Noriyuki Ebi, Seiji Doi, Hiroshi Ishii, Makoto Furugen, Jiro Fujita, Maako Ide, Junji Kishimoto, Isamu Okamoto, Masaki Fujita, Lung Oncology Group in Kyushu (LOGIK)

**Affiliations:** 1 Department of Respiratory Medicine, Fukuoka University Hospital, Fukuoka, Japan; 2 Department of Respiratory Oncology, Iizuka Hospital, Iizuka, Japan; 3 Department of Respiratory Medicine, Japan Community Health Care Organization, Isahaya General Hospital, Isahaya, Japan; 4 Department of Respiratory Medicine, Fukuoka University Chikushi Hospital, Chikushino, Japan; 5 First Department of Internal Medicine, University of the Ryukyus Hospital, Nakagami, Japan; 6 Research Institute for Diseases of the Chest, Graduate School of Medical Sciences, Kyushu University, Fukuoka, Japan; 7 Center for Clinical and Translational Research, Kyushu University Hospital, Fukuoka, Japan

**Keywords:** Lung cancer, febrile neutropenia, sitafloxacin, outpatient

## Abstract

**Introduction:**

Febrile episodes in patients with cancer and chemotherapy-induced neutropenia can be life-threatening and generally require prompt administration of broad-spectrum antimicrobials. However, little evidence exists for treating patients with solid tumors and febrile neutropenia (FN) with oral antimicrobials.

**Methods:**

In this prospective study, we aimed to determine the efficacy and safety of sitafloxacin (STFX) for treating FN in lung cancer patients. In this prospective study, low-risk FN patients with lung cancer received STFX. The primary endpoint was response rate, defined as 5 sequential days of absence of fever without adverse events. The study was registered as UMIN000010911.

**Results:**

As a result, STFX was administered to 26 patients, all of whom survived during its administration. Of the 26, 14 completed primary endpoint (53.85%). The low response rate was attributed to occurrence of fevers of unknown cause rather than failure of FN treatment. Only two patients received antibacterial agents other than STFX. If response rate omitted absence of fever and been defined only as recovery from FN without changing microbial agents or serious complications, the response rate would have been 91.67%. Adverse events occurred in eight patients, none of which were serious.

**Conclusions:**

In conclusion, STFX might be used to treat low-risk FN in patients with lung cancer; however, a more detailed study will be required in future.

## Introduction

Febrile episodes in cancer patients with chemotherapy-induced neutropenia can be life-threatening and are generally considered to require prompt administration of broad-spectrum antimicrobial agents. Such episodes have been defined as febrile neutropenia (FN). In cancer patients, FN is associated with considerable morbidity, mortality, and high medical costs ^(1)^. Excellent guidelines for FN have been published and updated by the Infectious Diseases Society of America ^(2)^. FN is characterized by the following: (i) polymorphonuclear neutrophil count of <500/μL, or neutrophils <1,000/μL with an expected drop to <500/μL; and (ii) temperature of >38.3°C at some stage or ≥38.0°C for 1 hour. In Japan, the second item of this definition of FN has been modified to a temperature of ≥37.5°C measured in the axillary fossa, as is the routine procedure in Japan ^(3)^. Although the Infectious Diseases Society of America guidelines do not differentiate between hematological and solid organ malignancies, they propose low- and high-risk categories for patients with FN ^(2)^. The purpose of this division is to enable treatment of low‐risk patients with oral antimicrobials in an outpatient setting, thus avoiding unnecessary hospitalizations, granulocyte colony-stimulating factor (G-CSF) use, and long, expensive courses of parenteral antibiotic therapy. Niho et al. reported that ciprofloxacin and clavulanate/amoxicillin treatment is effective against FN in low-risk patients ^(4)^. Levofloxacin (LVFX) has also been found to achieve excellent results ^(5)^. Cooper et al. reported that outpatient treatment with oral, single-agent, broad-spectrum fluoroquinolones achieves promising results against FN in low-risk patients ^(6)^.

The Multinational Association of Supportive Care in Cancer (MASCC) developed an internationally validated scoring system for identifying low-risk cancer patients with FN ^(7)^. Factors in this scoring system (MASCC score) comprise severity of disease, presence of hypotension, chronic obstructive pulmonary disease (COPD), dehydration, solid organ malignancy, history of fungal infection, outpatient care, and age younger than 60 years. MASCC score ≥ 21 is associated with low risk, the positive predictive value being 91%, specificity 68%, and sensitivity 71%. The higher the overall score, the greater the likelihood of fever resolution without any serious complications. FN in patients with lung cancer is considered to be low-risk unless the patient presents with COPD. However, whether all low-risk patients should be treated with oral antibiotics has not yet been determined.

Little evidence exists regarding the optimal type of antimicrobial agent for outpatients with low-risk FN and solid tumors. LVFX or ciprofloxacin are reportedly useful for low-risk FN ^(4)^. Sitafloxacin (STFX), a new quinolone antibacterial drug, was launched in Japan in 2008. Although LVFX has been recommended for low-risk group FN ^(3)^, STFX demonstrates stronger antibacterial activity against various pathogens than does LVFX ^(8)^. Therefore, we conducted this prospective study to determine the efficacy and safety of STFX for treating lung cancer patients with FN in an outpatient setting. The objective of this study was to determine the efficacy and safety of STFX for low-risk FN in lung cancer patients.

## Materials and Methods

### Patient eligibility

This study was done as a prospective study. The eligibility criteria were as follows: (i) provision of written informed consent to participate in the study; (ii) age ≥ 20 years; (iii) lung cancer accompanied by chemotherapy-induced neutropenia (polymorphonuclear neutrophil count of <1,000/μL); and (iv) temperature of ≥37.5°C. Body temperature was measured in the axillary fossa, as is routine practice in Japan. MASCC scores were evaluated, and an MASCC score ≥ 21 is considered to denote low risk ^(7)^.

Patients were excluded if they (i) received systemic antimicrobial agents for FN, (ii) received corticosteroid therapy, gamma-globulin therapy, or fluoroquinolones, (iii) presented with a history of renal failure (serum creatinine concentration of >2.0 mg/dl), or (iv) were pregnant or lactating. Granulocyte colony-stimulating factor and non-steroidal anti-inflammatory drugs were administered to patients with severe neutropenia at the discretion of that patient’s physician. Enrollment in the study was completed by faxing the entry form to the registration center at the Lung Oncology Group in Kyushu (LOGIK), where the eligibility criteria were checked.

This study was approved by the local Institutional Review Boards and was conducted in compliance with the guidelines of good clinical practice and the principles of the Declaration of Helsinki. All patients provided written informed consent prior to study entry (Clinical Research Network Fukuoka C13-6-03 on June 13, 2013). The study was registered as UMIN000010911.

### Clinical evaluation

The patients were clinically evaluated, including determination of their MASCC scores, and subsequently underwent daily examinations by an investigator at each center. The initial evaluation included a thorough medical interview and physical examination, complete blood cell count, urinalysis, blood chemistry profiles, and measurement of C-reactive protein. This evaluation was performed before commencing antimicrobial therapy (Day 0) and on Days 3 and 7. Samples for bacteriological examination were also obtained from all possible sites of infection on Day 0. Further bacteriological samples were obtained as indicated.

### Administration of study drug and antimicrobial therapy schedule

**Figure 1.**
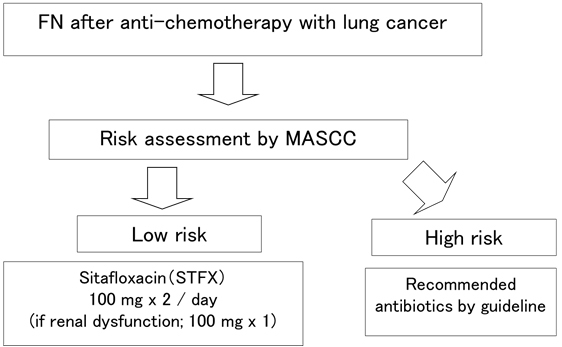
Flow chart of the study.

Once the criteria was fulfilled, STFX was initiated (Day 0) and continued for at least 5 days, unless the patient’s clinical condition worsened. If the fever persisted for more than 72 hours or recurred after an initial response, possible causes of fever were re-evaluated, and further antimicrobial therapy was selected at the discretion of each physician. Flow chart of the study was shown in Figure 1.

### Response to therapy

The primary endpoint of the present study was the rate of completing treatment, defined as 5 consecutive days of defervescence without any serious medical complications. The secondary endpoints were the rates of absence of fever at 72 hours and on Days 7 and 14, and safety. Relevant hematological and biochemical variables were evaluated, and urinalysis was performed on Days 0, 3, and 7 and on termination of therapy, more often if necessitated by the patient’s condition. Renal function was assessed on the basis of serum creatinine and hepatic function on the basis of serum bilirubin, transaminase, and alkaline phosphatase concentrations. The response rate was defined as the rate of recovery from FN without a change in microbial agent or serious complications.

### Statistical analysis

In this multi-institutional prospective study, the data required to assess the treatment response and incidence of adverse events were recorded. The primary endpoint was the rate of completion of treatment, defined as 5 consecutive days of defervescence without any serious medical complications.

LVFX and moxifloxacin, fluoroquinolone antimicrobial agents that are similar to STFX, have been reported as monotherapy for FN, being effective in 76.5% and 94.1% of cases, respectively ^(5), (6)^. The aim of this study was to determine whether treatment with STFX is superior to treatment with other fluoroquinolone antimicrobial agents, which we selected as a historical control. The threshold effective ratio for other fluoroquinolone antimicrobial agents was set at 76.5% and the expected effective ratio for FN at 90%. A normal approximation test with α = 0.05 (one-sided) determined that 50 cases would satisfy a detection power 1-β = 0.8. To allow for dropouts, the target number of cases was set at 54.

Two-sided confidence intervals (CIs) for chemotherapeutic responses were determined and χ^2^ testing performed using the JMP version 7.0 software program (SAS Institute, Cary, NC, USA) and the Wilson CI with no continuity correction.

## Results

### Characteristics of the study cohort

Between June 2013 and September 2017, 26 patients were enrolled in the present study. The study was stopped before the target number of 54 cases had been accumulated because of the unexpectedly long time taken to accrue participants. All participants were treated with STFX in the six institutions associated with LOGIK group. No patient died. The patients’ personal and clinical characteristics at the time of enrollment are summarized in Table 1. Demographic and clinical characteristics of FN are shown in Table 2.

**Table 1.** Personal and Clinical Characteristics of the Study Patients.

	N = 26
	Median or range
Sex	Male;19, Female;7
Age (years)	70.0 (50-82)
PS	0, 11; 1, 13; 2, 1; 3, 1; 4, 0
Creatinine (mg/dL)	0.93 (0.4-1.7)
Histology	Sm, 11; Ad, 11; Sq, 3; La, 1
Anticancer chemotherapy (line)	1^st^, 12; 2^nd^, 13; 3^rd^, 1
Comorbidity	No; 13, Yes; 13
COPD	No, 26; Yes, 0
Smoking status	No, 7; Ever, 13; Current 6
Brinkman index	1200 (150-1820)
MASCC score	21.0 (21-24)

Ad, adenocarcinoma; COPD, chronic obstruction pulmonary disease; MASCC, Multinational Association of Supportive Care in Cancer; La, large cell; PS, performance status; Sm, small cell; Sq, squamous cell. Brinkman index was shown among ever and current smokers. Comorbidity included as follows: diabetes mellitus, 6 cases; interstitial pneumonia, 3 cases; hypertension, 1 case; hyperlipidemia, 1 case; congestive heart failure, 1 case; old myocardial infarction, 1 case; bronchial asthma, 1 case; gastric cancer, 1 case; renal dysfunction, 1 case.

**Table 2.** Demographic and Clinical Characteristics of Febrile Neutropenia.

	Median	Range
WBC/ml	1125	300-2370
Neutrophils/ml	300	6-828
RBC × 10000/ml	310	115-453
Platelet × 10000/ml	11.0	1.7-36.2
CRP mg/dl	3.77	0.02-15.12

WBC; white blood cell, RBC; red blood cell, CRP; c-reactive protein.

### Clinical response

The rate of completion of primary endpoint (5 consecutive days of defervescence without any serious medical complications) was 53.85% (95% CI, 35.46%-71.24%). Of 26 patients, 14 completed primary endpoint. However, only two patients required antibacterial agents other than STFX, both of whom were treated successfully with meropenem. Moreover, two episodes of fever were identified as presented with causes other than infection, namely tumor fever and radiation pneumonitis. If we defined the response rate merely as recovery from FN without a change in microbial agent or serious complications, then the response rates would have been 91.67% (95% CI, 74.15%-97.68%).

Rates of absence of fever at 72 hours, 7 days, and 14 days were 53.84%, 86.36%, and 77.78%, respectively. Fever resolved by 72 hours in 14/26 patients (53.84%), by 7 days in 19/22 (86.36%), and by 14 days in 14/18 (77.78%).

### Adverse events

Adverse events occurred in 30.77% of the patients (95% CI, 16.50%-49.99%). These events included liver dysfunction, hyponatremia, renal dysfunction, and ataxia. According to the Common Terminology Criteria for Adverse Events, no grade 3 or higher adverse events were found. Renal dysfunction, hyponatremia, and liver dysfunction each persisted in one patient but did not progress. No patient developed diarrhea. The adverse events are presented in detail in Table 3.

**Table 3.** Details of Adverse Events.

Adverse event	Grade 1	Grade 2	Grade 3	Grade 4	Grade 5	Total
Ataxia		1				1
High serum creatinine	2					2
Hyponatremia	1					1
Liver dysfunction	1	3				4

*Grades 1-5 are as specified in the Common Terminology Criteria for Adverse Events v4.0.

### Microbiological response

In the present study, only a few patients presented with a microbiologically or clinically documented infection. Blood cultures were performed in 20 cases, only one of which was positive. This positive culture was for *Haemophilus* spp. Sputum cultures were performed in five cases; methicillin-sensitive *Staphylococcus aureus* was detected in one and *Enterobacter* in one.

## Discussion

Patients undergoing anti-tumor chemotherapy, including those with solid organ malignancies, frequently develop FN. Intravenous antimicrobial agent monotherapy is usually recommended; however, oral antimicrobials can be used if the FN is low risk. What type of antimicrobial agent should be used is yet to be determined. We investigated the role of intravenous antibiotics in FN in patients with lung cancer in past ^(9), (10), (11)^. We currently investigated the role of oral antibiotics, STFX in low-risk FN patients with lung cancer.

The rate of completion of primary endpoint (5 consecutive days of defervescence without any serious medical complications) was 53.85% (95% CI, 35.46%-71.24%). This low response rate was attributed not to failure of the treatment for FN but to fever of unknown cause, which occurred frequently in the form of abrupt onset of fever below 37.5°C. Only two patients required antibacterial agents other than STFX, both of whom were treated successfully with meropenem. Moreover, two episodes of fever were identified as demonstrating causes other than infection, namely tumor fever and radiation pneumonitis. These last two cases were not evaluated. If we defined the response rate merely as recovery from FN without a change in microbial agent or serious complications, then the response rates would have been 91.67% (95% CI, 74.15%-97.68%). Thus, we considered that our low response rate was due to the inclusion of 5 consecutive days of absence of fever in our definition of the primary endpoint.

Oral antibiotics are considered safe in patients with low-risk FN. Malik and colleagues performed a prospective study in which they randomized patients to receive oral ofloxacin 400 mg immediately and twice daily thereafter in hospital or as outpatients. They reported that 78% of inpatient and 77% of outpatient fevers resolved with no modification of the initial treatment ^(12)^. Niho and colleagues performed a prospective study comparing treatment with oral ciprofloxacin (200 mg) and amoxicillin-clavulanate (375 mg) administered every 8 h against treatment with intravenous ceftazidime (1 g) administered every 12 h in low-risk patients with FN and lung cancer. Treatment was successful without modification in 91% of the episodes in patients receiving the oral regimen and 79% of the episodes in patients receiving the intravenous regimen ^(4)^. Hidalgo and colleagues performed a randomized study comparing a combination of intravenous ceftazidime and amikacin with oral ofloxacin, and they found that patients recovered uneventfully in 91% of the episodes in the former (inpatient) group and 89% in the ofloxacin group ^(13)^. Freifeld and colleagues performed a randomized trial of oral ciprofloxacin plus amoxicillin-clavulanate versus intravenous ceftazidime in patients with FN during chemotherapy for cancer that were at low risk of complications. Treatment was successful without the need for modification in 71% of episodes in the oral therapy group and 67% of episodes in the intravenous therapy group ^(14)^.

Vedi and colleagues performed a meta-analysis and found that, in carefully selected low-risk children with FN, empirical treatment with oral antibiotics is a safe and effective alternative to intravenous antibiotics and decreases the cost of treatment and the psychosocial burden on these children and their families ^(15)^. A Cochrane study demonstrated that oral antibiotic treatment is an acceptable alternative to intravenous treatment in patients with cancer and FN (excluding patients with acute leukemia) who are hemodynamically stable and do not present with organ failure, pneumonia, infection of a central line, or severe soft-tissue infection ^(16)^. Another Cochrane study reported that treatment failure and mortality is probably equivalent for outpatient treatment and standard hospital (inpatient) treatment of low-risk FN in people with cancer, the former demonstrating the advantage of decreasing duration of hospitalization ^(17)^.

In this study, the response rates would have been 91.67% if we defined response rate simply as recovery from FN without a change of microbial agent or serious complications. The result was superior to historical controls, although registered number of cases could not attain. No serious complications occurred. According to the above-cited reports and the present findings, outpatient oral antibiotic therapy with oral fluoroquinolone, especially STFX, is safe and effective in patients with low-risk FN. Since the choice of antimicrobial agents is crucial for FN, the appropriate antimicrobial agents should be selected. Bacterial translocation in intestines might be important for occurrence for FN. STFX demonstrates stronger antibacterial activity against various pathogens than does LVFX ^(8)^, especially for anaerobes. Although LVFX had been widely prescribed for FN in Japan, this study provided important evidence that STFX could be selected for FN treatment.

The present study exhibited several limitations. The first limitation is the low rate of completion of treatment as a result of our definition of this primary endpoint as 5 consecutive days of absence of fever being too strict. It is worth noting that the definitions of the primary endpoint were unclear in other studies of FN ^(18), (19), (20)^. Therefore, we added an additional analysis in which the goal of treatment of FN was no change in the initial antimicrobial agent and no serious adverse events, with no mention of fever. This endpoint was achieved in 91.67% of our study patients. The second limitation of the present study was the small patient cohort; its small size made demonstrating statistically significant differences difficult. Additionally, we were unable to reach the required number of patients as determined before initiation of the study. One of the reasons for this failure was the frequent use of granulocyte colony-stimulating factor to prevent FN. Therefore, we were unable to analyze our findings statistically. Further large studies are required. However, the results of treatment of FN with STFX in this study are useful.

In summary, we conducted a prospective study to determine the efficacy and safety of STFX in treatment of low-risk FN in lung cancer patients. Although the present study cohort was small, our results indicate that STFX monotherapy might be effective against low-risk FN in lung cancer patients for the first time in the world. However, more detailed study will be required in future.

## Article Information

### Conflicts of Interest

None

### Sources of Funding

This study was performed using own funds. This research received no specific grant from any funding agency in the public, commercial, or not-for-profit sectors.

### Acknowledgement

We thank Dr Trish Reynolds, MBBS, FRACP, from Edanz Group (https://en-author-services.edanz.com/ac) for editing a draft of this manuscript. We also thank the Clinical Research Support Center, Kyushu for managing this study.

### Author Contributions

All authors meet the ICMJE authorship criteria. All authors belong to LOGIK. RO, TM, and MF conceived and carried out the clinical study, and they prepared the manuscript. NE, SD, HI, FM, JF, and MI cooperated with accumulation of clinical data. KJ perform statistical analysis and advised study protocols. IO organized the clinical study. All authors read and approved the final manuscript.

### Approval by Institutional Review Board (IRB)

C13-6-03 on 13 June 2013, Clinical Research Network Fukuoka.

The study was registered as UMIN000010911.

### Informed Consent

Informed consent was obtained from all subjects involved in the study.

### Data Availability Statements

The data presented in this study are available on request from the corresponding author. The data are not publicly available due to lack of informed consents for data availability.
